# Cognitive Impairment and Its Associated Risk Factors in the Elderly With Type 2 Diabetes Mellitus

**DOI:** 10.3389/fpsyt.2021.669725

**Published:** 2021-10-21

**Authors:** Rosnadia Suain Bon, Suthahar Ariaratnam, Zanariah Mat Saher, Mariam Mohamad, Fatt Soon Lee

**Affiliations:** ^1^Department of Psychiatry, Faculty of Medicine, Universiti Teknologi MARA, Cawangan Selangor, Kampus Selayang, Selangor, Malaysia; ^2^Psychiatry Unit, Faculty of Medicine and Defence Health, National Defence University of Malaysia, Kuala Lumpur, Malaysia; ^3^Department of Psychiatry and Mental Health, Hospital Kuala Lumpur, Kuala Lumpur, Malaysia; ^4^Department of Public Health, Faculty of Medicine, Universiti Teknologi MARA, Cawangan Selangor, Kampus Sungai Buloh, Sungai Buloh, Malaysia; ^5^Department of Medicine, Hospital Kuala Lumpur, Kuala Lumpur, Malaysia

**Keywords:** cognitive impairment, elderly, type 2 diabetes mellitus, depression, anxiety

## Abstract

Cognitive impairment is not only common but may also act as a precursor for dementia. Moreover, diabetes mellitus has been shown to promote the progression of dementia. This study aims to determine the prevalence of cognitive impairment and its associated risk factors among the elderly patients having type 2 diabetes mellitus (T2DM) in Hospital Kuala Lumpur, Malaysia. This is a cross-sectional study involving 113 participants who were attending the physician clinic. The participants were selected using the universal sampling method. The tools included a sociodemographic questionnaire, the Montreal Cognitive Assessment, the Depression Anxiety Stress Scale, and the Mini-International Neuropsychiatry Interview. Descriptive analysis was performed and followed by multiple logistic regression. The prevalence of cognitive impairment, depressive disorder, and anxiety disorder was 46.9, 10.6, and 2.7%, respectively. Indians (aOR = 4.90, 95% CI = 1.57–15.27) as well as participants who had completed their secondary (aOR = 0.34; 95% CI = 0.12–0.96) and tertiary (aOR = 0.05; 95% CI = 0.01–0.26) levels of education were significantly associated with cognitive impairment. The prevalence of cognitive impairment was high as well as significantly associated with the ethnicity and education levels of the elderly participants. Indeed both secondary and tertiary education decreased the odds ratio of developing cognitive impairment when compared with no formal and primary education. To determine and reduce the potential risk factors which contribute to cognitive impairment, an early diagnosis of T2DM is crucial. The early detection and treatment of T2DM as well as its associated risk factors are key factors in protecting against cognitive impairment.

## Introduction

The percentage of the elderly population has been on the increase globally. According to the World Health Organization (1), the number of people aged 60 years and older is expected to rise from 900 million in 2015 to 2 billion in 2050, an increase of 12 to 22% within a span of 35 years. This category of population is often associated with multiple medical comorbidities as well.

Type 2 diabetes mellitus (T2DM) has been identified as one of the established risk factors for both cognitive impairment (2) and cardiovascular diseases. The Malaysian National Health and Morbidity Survey 2015 (3) found a 17.5% prevalence of diabetes mellitus in adults more than 18 years of age. There was an increasing trend in prevalence with increasing age, from 5.5% in the 18–19 years age group to 39.1% among the 70–74 years age group.

Research on depression and anxiety in T2DM patients has been robust (4–6). Regrettably, these investigations have only used screening tools to establish depression and anxiety. The use of a diagnostic criterion to ascertain the accurate prevalence of depression and anxiety in patients is scarce. Nevertheless, a study in Taiwan by Huang et al. (7) using diagnostic tools only determined the prevalence of generalized anxiety disorder among T2DM patients.

Cognitive impairment can range from loss of function in multiple mental abilities such as concentration, memory recall, reasoning, learning, and decision making (8). A combination of cognitive impairment and a medical condition such as T2DM might be severe. As a result, patients may ignore treatment care by failure to follow up on appointments. Medical literature has shown that T2DM patients are more susceptible to cognitive impairment with advancing age. Additionally, structural and physiological deterioration associated with an aging brain appeared to be accelerated in the elderly with T2DM (9). Consequently, the failure of these patients in seeking timely and scheduled treatment would only accelerate the cognitive decline.

With a growing aging population along with the rising prevalence of diabetes, cognitive impairment would steadily increase as well. Furthermore, cognitive impairment has been a harbinger of neurocognitive disorders. Therefore, it is only reasonable to identify the risk factors in these individuals so that intervention could begin early before its progression into a full-blown neurocognitive disorder. Early detection would certainly help reduce the human as well as socio-economic liability in the medical field and ultimately alleviate caregiver burden.

The purposes of this study were to determine the prevalence of cognitive impairment, depression, anxiety, and its associated risk factors in the elderly with T2DM using diagnostic criteria.

## Methodology

A cross-sectional study using universal sampling was conducted at the General Internal Medicine and Endocrine specialist clinics of Hospital Kuala Lumpur, which is the largest hospital under the Ministry of Health in Malaysia.

The sample size was calculated based on the expected prevalence of mild cognitive impairment of 64.7% among the elderly with a medical illness in a local study (10). Hence, the sample size was determined using the following formula (11):

$n={\left(Z_{1-\alpha }\right)}^{2}\left[P\left(1-P\right)/D^{2}\right]$

*Z*1–α = *Z*0.95 = 1.96 (for CI of 95%, *Z* = 1.96)
*P* = 64.7% (prevalence from the local study)
*D* (absolute precision required) = 5%
*n* = 1.96^2^ [0.06 (1–0.06)/0.05^2^] = 91

Sample size required:

*n* = 86.67 + 20% (non-response) = 109.

Thus, the minimum sample size planned for this study was 109.

This study population included patients aged 60 and older who were diagnosed with T2DM. Only patients who had consented to the study were enrolled in this assessment.

Approval to conduct the study was obtained from the Ethical Board of Research Committee of the Universiti Teknologi MARA (UiTM), the Clinical Research Center of Hospital Kuala Lumpur, and the National Medical Research Registry.

Firstly, the participants were provided with self-administered questionnaires, in Malay version, which probed the sociodemographic factors (age, gender, ethnicity, marital status, education level, living status, and smoking habit) and clinical variables [height and weight, duration of diabetes, HbA1c level, medication for diabetes, hyperlipidemia, triglyceride, high-density lipoprotein (HDL), hypertension status, number of medications for hypertension, and medical comorbidities] of the patients.

Body mass index (kg/m^2^) was classified based on the Malaysian Clinical Practice Guideline (12). Obesity can be categorized as normal (18.5–22.9), overweight (>23), pre-obese (23–27.4), obese I (27.5–34.9), obese II (35–39.9), and obese III (>40).

Secondly, the participants were required to complete the Malay versions of the Montreal Cognitive Assessment (10) and the Depression, Anxiety, Stress Scale-21 (DASS-21-BM) questionnaires (13).

The participants were screened for cognitive impairment using the Montreal Cognitive Assessment (MoCA) tool, which is a brief cognitive screening test. It has 30 questions that help to assess how the cognitive function of an individual is affected. It evaluates different types of cognitive abilities such as orientation, short-term memory, executive function, language abilities, abstraction, animal naming, attention, and clock-drawing test. The scores on the MoCA ranged from 0 to 30, with a score of 26 and higher generally considered normal. A total score below 26 indicates cognitive dysfunction.

DASS 21, a self-report screening tool, was used to identify depression and anxiety. It has 21 items which are tailored to assess the symptoms of depression and anxiety, respectively. For each question, the patients reported their experiences on each symptom, ranging from “0” (does not apply to me) to “3” (applies to me most of the time) on a four-point severity scale (Likert scale). The scores for each item (depression, anxiety, and stress) were added, respectively, to give a total score. For depression, the total score was categorized as follows: normal (0–9), mild (10–13), moderate (14–20), severe (21–27), and extremely severe (more than 28). For anxiety, the total score was categorized as normal (0–7), mild (8 to 9), moderate (10–14), severe (15–19), and extremely severe (more than 20).

Subsequently, the participants who had been classified as at least a mild category of depression and anxiety from the DASS-21-BM scale were subjected to the Malay version of the Mini-International Neuropsychiatric Interview (M.I.N.I.) (14). The M.I.N.I. is a brief, diagnostic, structured interview based on the Diagnostic and Statistical Manual of Mental Disorders (DSM-IV) (15) that determines psychiatric disorders such as depressive disorder, generalized anxiety disorder, and panic disorder without agoraphobia.

The data were analyzed using the International Business Machines (IBM®) Statistical Package for Social Sciences (SPSS version 21) computer program (IBM®, 2012). The socio-demographic and clinical variables were described as frequency (%) for categorical data and mean (standard deviation, SD) for continuous data. Similarly, the prevalence of cognitive impairment, depression, anxiety, and stress was described as frequency (%).

Logistic regression was conducted to examine the association between independent and dependent variables. From the simple logistic regression analysis, independent variables that had a *p* < 0.25 were included in the multiple logistic regression model (16). The “ENTER” method was used to investigate all the associated factors with cognitive impairment. The multiple logistic regression model was able to establish the association between the independent variables to a dichotomous dependent variable (cognitive impairment). Controlling the confounders allowed the predictors or associated risk factors of cognitive impairment to be identified. Significant factors associated with cognitive impairment were checked for interaction and multi-collinearity with variance inflation factor. Subsequently, the Hosmer–Lemeshow test was used to assess the goodness-of-fit for logistic regression.

## Results

A total of 113 patients participated in this study. Table 1 summarizes the sociodemographic and clinical data of the study participants.

**Table 1:** Sociodemographic and clinical data of participants (*N* = 113).

**Characteristic**		***n* (%)**	**Mean (SD)**
Age			68.4 (6.0)
Gender	Male	57 (50.4)	
	Female	56 (49.6)	
Ethnicity	Malay	58 (51.3)	
	Chinese	19 (16.3)	
	Indian	36 (31.8)	
Marital status	Single	9 (8)	
	Married	100 (88.5)	
	Divorced	4 (3.5)	
Educational level	No formal education	8 (7.1)	
	Primary	43 (38.1)	
	Secondary	41 (36.3)	
	Tertiary	21 (18.6)	
Employment status	Never employed	28 (24.8)	
	Employed	25 (22.1)	
	Pensioner/retired	60 (53.1)	
Living status	Alone	7 (5.3)	
	Spouse	61 (54.0)	
	Children	40 (35.4)	
	Sibling	5 (4.4)	
	Nursing home	1 (0.9)	
Smoking	Yes	11 (9.7)	
	No	102 (90.3)	
BMI	Normal	22 (19.5)	
	Abnormal	91 (80.5)	
Duration DM	Less than 20 years	2 (1.8)	
	More than 20 years	111 (98.2)	
HBA1c Level	Normal	22 (19.5)	
	Abnormal	91 (80.5)	
Medication DM	None	9 (8)	
	OHA	39 (34.5)	
	Insulin and combined	65 (57.5)	
Compliance	Yes	70 (61.9)	
	No	43 (38.1)	
Hyperlipidemia	Yes	111 (98.2)	
	No	2 (1.8)	
TG level	Normal	81 (71.7)	
	Abnormal	32 (28.3)	
HDL level	Normal	90 (79.6)	
	Abnormal	23 (20.4)	
Hypertension	Yes	102 (90.3)	
	No	11 (9.7)	
HPT medication	Yes	102 (90.3)	
	No	11 (9.7)	
Number of HPT medication	Less than 2	91 (80.5)	
	3 or more	22 (19.5)	
Number of comorbidities	1	2 (1.8)	
	More than 2	111 (98.2)	
Medical comorbidities	Cardiovascular	102 (37)	
	Endocrine	4 (1.5)	
	Rheumatology	7 (2.6)	
	Nephrology	29 (10.7)	
	Gastroenterology	30 (11.1)	
	Neurology	6 (2.22)	
	Respiratory	35 (13)	
	Oncology	7 (2.6)	
	Gynecology	5 (1.9)	
	Ophthalmology	5 (1.9)	
	Orthopedic	6 (2.2)	
	Urology	30 (11.1)	
	Hematology	4 (1.5)	

*BMI, body mass index; HPT, hypertension; TG, triglyceride; HDL, high-density lipoprotein*.

Gender distribution was 50.4% male and 49.6% female. The mean age of the participants was 68.4 years (SD = 6.0). The participants comprised 51.3% Malays, 31.8% Indians, and 16.3% Chinese. Marital status profiling revealed that majority were married (88.5%), while the single and divorced participants were 8 and 3.5%, respectively. Pertaining to educational level, 74.4% of the participants had completed primary (38.1%) and secondary (36.3%) levels of education, while 7.1 and 18.6% had no formal education and completed tertiary level of education, respectively. As for the employment status, majority were retired/pensioner (60%). A high percentage of the participants (94%) lived with their family (either with spouses, children, or siblings), and 6% lived without families (either in nursing homes or alone). Most of the participants were non-smokers (90%).

As shown in Table 1, 80.5% registered abnormal BMI, 98% had been diagnosed with T2DM for more than 20 years, 80.5% had abnormal HbA1c, 57.5% were on insulin treatment or combined medication for T2DM, 61.9% were in compliance to their treatment schedule, 98.2% had hyperlipidaemia, 71.1% had normal triglyceride levels, 79.6% had normal HDL levels, 90.3% had hypertension, 80.5% had used <2 types of medication, 98.2% had two or more medical comorbidities, and 37% had cardiovascular diseases.

Table 2 illustrates the prevalence of cognitive impairment, depression, anxiety, and stress as 46.9, 11.5, 7.1, and 0.9%, respectively.

**Table 2:** Prevalence of cognitive impairment, depression, and anxiety.

**Items**	***n***	**%**
Cognitive impairment	53	46.9
Depression	13	11.5
Anxiety	8	7.1

*The cut-off score of 23 was used for the Montreal Cognitive Assessment. Cut-off score of 10 and above for depression and score of 8 or more for anxiety were used for Depression and Anxiety symptoms respectively using the Malay version of DASS 21. T2DM, Type 2 Diabetes Mellitus*.

Table 3 depicts that 10.6% of the participants had depressive disorders, while 0.9% had panic disorders without agoraphobia and 1.8% had generalized anxiety disorders.

**Table 3:** Prevalence of depressive and anxiety disorders.

**Diagnosis**	***n***	**(%)**
Depressive disorder	12	10.6
Panic disorder without agoraphobia	1	0.9
Generalized anxiety disorder	2	1.8

Table 4 demonstrates that Indians (adjusted OR = 4.90, 95% CI = 1.57–15.27; *p* = 0.006) as well as participants who had completed their secondary (adjusted OR = 0.34; 95% CI = 0.12–0.96; *p* = 0.040) and tertiary (adjusted OR = 0.05; 95% CI = 0.01–0.26; *p* = 0.001) levels of education were significantly associated with cognitive impairment in the elderly with T2DM.

**Table 4:** Factors associated with cognitive impairment in the elderly with T2DM using logistic regression (*N* = 113).

**Variables**		**Crude OR^a^ (95% CI)**	**Adjusted OR^b^ (95% CI)**	**Wald statistic (df)**	***p*-value**
Age		0.947 (0.88, 1.01)	0.93 (0.859, 1.01)	2.96 (1)	0.085
Gender	Male	1	1		
	Female	2.29 (1.08, 4.86)	1.26 (0.39, 4.11)	0.15 (1)	0.695
Ethnicity	Malay	1	1		
	Chinese	0.39 (0.17, 0.91)	1.28 (0.37, 4.47)	0.15 (1)	0.696
	Indian	0.57 (0.19, 1.76)	4.90 (1.57, 15.27)	7.48 (1)	0.006
Education level	No formal education and primary	1	1		
	Secondary	0.32 (0.14, 0.75)	0.34 (0.12, 0.96)	4.13 (1)	0.042
	Tertiary	0.08 (0.22, 0.32)	0.05 (0.01, 0.26)	12.08 (1)	0.001
Employment status	Never Employed	1	1		
	Employment	0.389 (0.16, 0.94)	0.65 (0.19, 2.28)	0.43 (1)	0.511
Living status	No family	1	1		
	Family	0.331 (0.06, 1.78)	0.22 (0.03, 1.61)	2.21 (1)	0.138
Compliance	No	1.000	1.000		
	Yes	0.652 (0.30, 1.40)	0.69 (0.27, 1.18)	0.54 (1)	0.459
Level HDL	Normal	1	1		
	Abnormal	2.034 (0.80, 5.18)	3.39 (0.98, 11.68)	3.74 (1)	0.053
Hypertension	No	1	1		
	Yes	0.469 (0.13, 1.70)	0.37 (0.072, 1.93)	1.38 (1)	0.239
Medication HPT number	Less than 2	1	1		
	3 or more	0.457 (0.17, 1.23)	0.31 (0.09, 1.13)	3.12 (1)	0.077

*95%CI, 95% confidence interval; T2DM, type 2 diabetes mellitus*.

a *Simple logistic regression*.

b *Multiple logistic regression*.

## Discussion

To the best of our knowledge, this is the first study to evaluate cognitive impairment specifically in the elderly population with T2DM in Malaysia using diagnostic criteria.

In our study, the prevalence of cognitive impairment among the elderly with T2DM in a hospital setting was 46.9%. This finding was similar to studies conducted in France (17), Nigeria (18), India (19), and Japan (20).

Using the diagnostic method, the prevalence of depressive disorder was 10.6%, while 2.7% had anxiety disorders (comprising panic and generalized anxiety disorders). This finding was comparable to that of a meta-analysis which had described an overall prevalence of depression as 10.9% using structured or semi-structured diagnostic interview methods (21). However, using the same technique of diagnosis, the prevalence of anxiety disorder appeared to be lower in our study compared to a collaborative study involving 15 nations (18%) (22) and the Baltimore Epidemiologic Catchment area (8.9%) (23). We theorize that this discrepancy might be due to the variations in the study design, measuring tools used, sample size as well as duration or severity of T2DM among the participants.

Our study demonstrated a significant association between ethnicity and education levels to cognitive impairment in the elderly with T2DM. Indians were five times more at risk of developing cognitive impairment (adjusted OR = 4.90; 95% CI = 1.57–15.27; *p* = 0.006) compared to Malays. For participants who had completed secondary education, they had decreased odds of developing cognitive impairment by 66% (adjusted OR = 0.34; 95% CI = 0.12–0.96; *p* = 0.042), while participants with a tertiary education background had decreased odds of developing cognitive impairment by 95% (adjusted OR = 0.05; 95% CI = 0.01–0.26; *p* = 0.001) compared to no formal and primary education.

Multiple studies demonstrated different rates of prevalence of cognitive impairment in T2DM across ethnicity—for instance, a cohort study of a population of elderly persons in a multi-ethnic community of Northern Manhattan, New York, found that cognitive impairment dementia (CID) in T2DM was more prevalent among African Americans and Hispanics when compared to Whites (11.4 vs. 4.9%; *p* = 0.06). This finding was obtained after age, gender, education, and apolipoprotein E4 allele were controlled. The hazard ratio relating T2DM and CID was 1.63 (95% CI, 1.26–2.09) (24). A study in Singapore discovered that vascular dementia was more common among Chinese and Malays, while Alzheimer's disease was more common in Indians and Eurasians (25).

Arvanitakis and colleagues (2) affirmed that T2DM is one of the established risk factors for cognitive impairment. The Malaysian National Health and Morbidity Survey, conducted in 2015, found 22.1% of Indians to have diabetes mellitus, which was the highest prevalence recorded. Consistent with the finding of this survey, our study has shown that cognitive impairment was prevalent among Indians compared to other races.

The association between levels of education and risk of developing cognitive impairment had been actively studied. Research on this subject matter proposed that education may act by raising the educational reserve and provide protection against dementia or cognitive impairment. Furthermore, individuals who had begun with more educational reserves take a longer time to reach the critical threshold for cognitive decline to commence (26). Besides this, another study found that individuals without formal education were associated with a higher prevalence of dementia (27). Additionally, a local study disclosed that mild cognitive impairment was significantly associated with low education level in patients with underlying medical comorbidities (10).

Our study has some potential limitations. Firstly, there was a potential selection bias. This single-center study was conducted at Hospital Kuala Lumpur, situated in an urban area that served a predominantly urban population who had good access to medical care. Secondly, being a cross-sectional survey, our study did not allow for cause and effect associations to be examined.

## Conclusions

Understanding cognitive impairment in T2DM patients, especially in the elderly population, is very crucial. Many factors, such as low education levels (those having no formal or secondary level of education) and ethnicity, particularly Indians, contribute to cognitive impairment in the elderly with T2DM. Therefore, identifying and managing these vulnerable individuals from the onset would certainly reduce the risk of developing cognitive impairment. A multidisciplinary approach involving physicians, psychiatrists, and endocrinologists is required to best meet the needs of each patient.

## Data Availability Statement

The data that support the findings of this study are available upon formal request. The data are not publicly available due to privacy concerns.

## Ethics Statement

The studies involving human participants were reviewed and approved by Ethical Board of Research Committee of Universiti Teknologi MARA (UiTM), Clinical Research Center (CRC) of Hospital Kuala Lumpur, and National Medical Research Registry (NMRR). The patients/participants provided their written informed consent to participate in this study.

## Author Contributions

RS, SA, and ZM: conceptualization. RS and FL: data collection. MM: formal analysis and software. RS, SA, ZM, MM, and FL: writing, review, and editing. All authors contributed to the article and approved the submitted version.

## Conflict of Interest

The authors declare that the research was conducted in the absence of any commercial or financial relationships that could be construed as a potential conflict of interest.

## Publisher's Note

All claims expressed in this article are solely those of the authors and do not necessarily represent those of their affiliated organizations, or those of the publisher, the editors and the reviewers. Any product that may be evaluated in this article, or claim that may be made by its manufacturer, is not guaranteed or endorsed by the publisher.
